# Vaginal neutrophil infiltration is contingent on ovarian cycle phase and independent of pathogen infection

**DOI:** 10.3389/fimmu.2022.1031941

**Published:** 2022-12-08

**Authors:** M. C. Latorre, C. Gómez‐Oro, I. Olivera‐Valle, E. Blazquez‐Lopez, J. Gallego‐Valle, A. Ibañez‐Escribano, P. Casesnoves, C. González‐Cucharero, M. A. Muñoz‐Fernandez, L. Sanz, J. Vaquero, P. Martín‐Rabadań, F. Perez‐Milan, M. Relloso

**Affiliations:** ^1^Laboratorio de InmunoReproduccion, Instituto de Investigación Sanitaria Gregorio Marañón, Madrid, Spain; ^2^Hepatología-Servicio de Aparato Digestivo, Hospital General Universitario Gregorio Marañón, Red de Enfermedades Hepáticas y Digestivas (CIBEREHD), Instituto de Salud Carlos III (ISCIII), Madrid, Spain; ^3^Laboratorio de InmunoRegulacion, Instituto de Investigación Sanitaria Gregorio Marañón, Madrid, Spain; ^4^Departamento de Microbiología y Parasitología, Facultad de Farmacia, Universidad Complutense de Madrid (UCM), Madrid, Spain; ^5^Laboratorio InmunoBiologia Molecular, Instituto de Investigación Sanitaria Gregorio Marañón, Madrid, Spain; ^6^Molecular Immunology Unit, Biomedical Research Institute Hospital Universitario Puerta de Hierro Majadahonda, Madrid, Spain; ^7^Servicio de Microbiología Clínica y Enfermedades Infecciosas, Hospital General Universitarion Gregorio Marañón (HGUGM), Madrid, Spain; ^8^Servicio de Obstetricia y Ginecología, Hospital General Universitario Gregorio Marañón, Madrid, Spain

**Keywords:** neutrophil infiltration, insemination, *C. albicans*, *T. vaginalis*, *N. gonorrhoeae*, HSV-2

## Abstract

The mucosa of the female reproductive tract must reconcile the presence of commensal microbiota and the transit of exogenous spermatozoa with the elimination of sexually transmitted pathogens. In the vagina, neutrophils are the principal cellular arm of innate immunity and constitute the first line of protection in response to infections or injury. Neutrophils are absent from the vaginal lumen during the ovulatory phase, probably to allow sperm to fertilize; however, the mechanisms that regulate neutrophil influx to the vagina in response to aggressions remain controversial. We have used mouse inseminations and infections of *Neisseria gonorrhoeae*, *Candida albicans*, *Trichomonas vaginalis*, and HSV-2 models. We demonstrate that neutrophil infiltration of the vaginal mucosa is distinctively contingent on the ovarian cycle phase and independent of the sperm and pathogen challenge, probably to prevent sperm from being attacked by neutrophils. Neutrophils extravasation is a multi-step cascade of events, which includes their adhesion through selectins (E, P and L) and integrins of the endothelial cells. We have discovered that cervical endothelial cells expressed selectin-E (SELE, CD62E) to favor neutrophils recruitment and estradiol down-regulated SELE expression during ovulation, which impaired neutrophil transendothelial migration and orchestrated sperm tolerance. Progesterone up-regulated SELE to restore surveillance after ovulation.

## Graphical Abstract

**(A)** Neutrophils infiltrate tissues following circadian rhythms as part of normal repair, as microbiota regulation or as immune-surveillance in the absence of inflammatory stimuli. **(B)** Some tissue infiltration is independent of circadian rhythms like intestine, liver and white adipose tissue. However, inflammatory signals up-regulate neutrophil extravasation in the tissues. Cervical tissue mediates an exceptionally high and continuous neutrophil infiltration in absence of inflammatory stimuli (diestrus 2 to 4 days) to maintain constant neutrophil immune-surveillance and control of commensal microbiota balance. However, during proestrus (12 to 20h) and ovulation (12 to 20h dark period), estradiol **(C)** induces neutrophil withdraw from the vaginal mucosa **(D)** defense to favor reproduction at the cost of a potential impact in the onset of STIs. Surprisingly, vaginal challenges do not induce increase neutrophil infiltration neither during the high number of neutrophil (metestrus) nor the low neutrophil (estrus) content stages in the vaginal lumen.
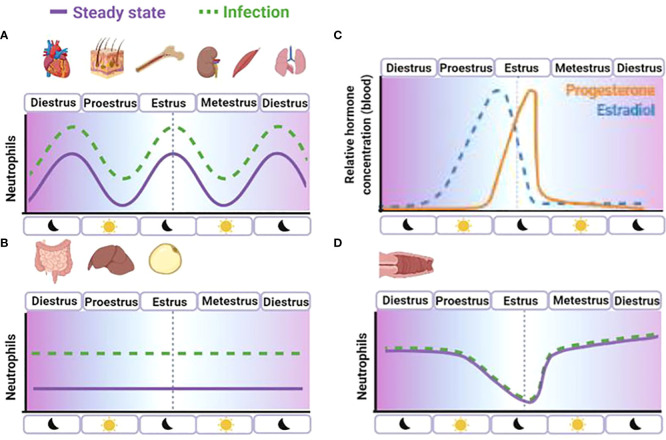

## Introduction

Multiple types of bacteria, viruses, fungi, and parasites are transmitted through sexual contact. Among these, several pathogens have a high prevalence in the population of which only some are currently curable (*Treponema pallidum*, *Neisseria gonorrhoeae*, *Chlamydia trachomatis* and *Trichomonas vaginalis*), whereas others are incurable viral infections with only symptomatic treatment (hepatitis B virus (HBV), herpes simplex virus (HSV), human immunodeficiency virus (HIV), human papillomavirus (HPV)). The cases of sexually transmitted infections (STI) and microorganism resistance increase yearly (CDC STD surveillance), and STIs have a substantial impact on the population at reproductive age, with overwhelming economic effects, working hours lost, and serious health consequences (infertility) beyond the infection itself (WHO STI surveillance 2015). Indeed, the World Health Organization (WHO) estimated that more than 1 million people acquire STIs worldwide every day despite all prevention efforts. Therefore, there is an unmet need to understand the interplay between pathogens causing STIs and host immune responses in order to design more efficient prevention strategies.

The female reproductive tract is highly susceptible to sex hormone variations during the menstrual cycle, which comprises three main phases: (a) follicular phase (proestrus in mice), (b) ovulatory or estrus, and (c) luteal phase (metestrus). Estradiol (E2) increases during proestrus, reaches a peak during the estrus, and gradually falls during the end of estrus. After ovulation, progesterone (P4) levels peak and lower during metestrus. Hormonal level fluctuations during the menstrual cycle have well-known effects on tissue-resident leukocytes (1–4), including neutrophils, the predominant phagocytic immune cells in the vaginal lumen.

Neutrophils are essential players in the innate immune response to invading bacteria (5), unicellular parasites (6), and fungi (7). In steady-state conditions, neutrophil trafficking is tightly controlled by circadian rhythms, as aberrant leukocyte recruitment contributes to tissue damage or inflammatory diseases (8). In response to infections or injuries, however, neutrophils are rapidly recruited to the site of inflammation to clear pathogens (9). Neutrophil recruitment to the tissues require adhesion and transmigration through endothelial cells walls. The initial process is the rolling of neutrophils which starts with the tethering of PSGL-1 and CD44 with the endothelial selectins E and P (SELE and SELP) on the EC, which mediate neutrophil rolling on EC. This is followed by the firm interaction between EC integrins (ICAM1 and VCAM1) with the neutrophil ligands (CD11a and CD49d) which ultimately allows extravasation into tissues [for review (10)]. Vaginal lumen typically contains neutrophils to maintain vaginal immune surveillance and STI protection. Importantly, neutrophils, which are very efficient at killing exogenous sperm, disappear from the vaginal lumen during the ovulatory phase facilitating exogenous spermatozoa survival and transit (11, 12). How vaginal mucosa deals with pathogens during ovulation to avoid inflammatory reactions that may harm sperm quality and cause infertility (13, 14) is still unknown. Here, we show that neutrophils are constitutively and highly recruited to the vaginal lumen regulated by sex hormones but independent of infection stimulus or insemination, globally favoring reproduction over the cost of increasing the risk of onset of STIs.

## Materials and methods

### Animals, vaginal cytology, and *in vivo* hormonal treatment

The IiSGM Animal Care and Use Committee and Comunidad de Madrid approved all the animal procedures (PROEX-188/18 and 198/19).

Eight week-old female BALB/c (H-2d) mice were maintained with environmental enrichment, under specific pathogen-free, 12h light/dark, temperature and humidity-controlled conditions in the Animal Facility of IiSGM. To determine the ovarian cycle stages, we gently placed 5μL of PBS on the vaginal entrance, flushed, collected, and observed under a light microscope (15).

To mimic the female ovarian cycle, mice previously treated with hormones were bilaterally ovariectomized under anesthesia (16). After a two-week recovery, females were injected subcutaneously with 0.006mg of 17β-E2 (Calbiochem, Germany) dissolved in 100µl of sesame oil (Sigma-Aldrich, USA) for 48h. Then, mice were treated with 0.2mg of P4 (Calbiochem, Germany) or 17β-E2 for 12h. After that, mice were challenged with 2×10^6^ *C. albicans* blastoconidia in 20µl of PBS into the vagina (17, 18). Finally, vaginal samples were obtained 12h later.

### Culture of microorganisms

*Candida albicans* (ATCC^®^ MYA­2876™) strain was grown on Sabouraud dextrose chloramphenicol agar plates (Conda, Spain) overnight at 30°C prior to the experiments (19). *Trichomonas vaginalis* (ATCC^®^C-1:NIH) was grown on TYM medium for 48h at 37°C in a 5% CO_2_ atmosphere prior to the experiments (20). The strain of *Neisseria gonorrhoeae* (ATCC^®^ 700825™) was grown on CG agar plates at 35-37°C in a 5% CO2 atmosphere for 48h (21). The stock of HSV-2 strain 333, was prepared and titrated by plaque assay in Vero cells (ATCC^®^ CCL-81™, Manassas, VA, USA) and stored at −80°C (22).

### Sperm collection

Mouse sperm were collected from CD1 Crl : CD1(ICR) (outbred) vasa deferens (16).

### Vaginal and peritoneal inoculations

To control the circadian rhythm regulation mice were selected by vaginal smear at the end of the dark stage (1h before the light switch on) which is the end of estrus (12 to 20h dark stage) and beginning of metestrus (12 to 20h light stage). Mice were inoculated into the vagina (20µl) or the peritoneum (100µl) with *Neisseria gonorrhoeae* (2×10^6^ ufc) (21), *Trichomonas vaginalis* (2×10^6^ trichomonads) (20), *Candida albicans* (2×10^6^ ufc) (19), mouse sperm (2×10^6^) (11, 16, 18), or HSV-2 (2×10^4^ pfu) (22) in PBS or PBS alone. After 2h of challenge mice were sacrificed and also checked their hormonal stage by vaginal smear.

### Vaginal and peritoneal secretions collection

Peritoneal cells were harvested by flush with 5ml of PBS into the peritoneal cavity (23). Vaginal secretions were gently collected by flushing the vagina four times with 50 µl of sterile PBS (16, 19). In both cases flushed medium was centrifuged, re-suspend in PBS 5% FBS and cells were collected at 4°C for analysis.

### Cellular phenotypic analysis by flow cytometry

Cellular phenotypic analysis was carried out by direct immunofluorescence with the following antibodies: CD11b M1/70 (eBioscience), Ly6g 1A8 (eBioscience) F4/80 BM8 (Biolegend), MHCII 2G9 (Biosciences), NK1.1 S17016D (Biolegend), CD3 145-2C11 (eBioscience), CD31 390 (Biolegend), αSMA (α smooth muscle actin) 1A4 (eBioscience), CD62-E REA369 (Miltenyi Biotec), CD62P RMP-1 (Biolegend), CD54 (Biolegend), CD106 (Biolegend), CD192 (CCR2) REA538 (Miltenyi Biotec)., PSGL-1 34 RA10 (Thermofisher), CD44 IM7.8.1 (Miltenyi Biotec), CD11a 121.7 (Thermofisher), CD49d 9C10 MFR4.B (Biolegend). All incubations were done in the presence of 50 μg/ml mouse IgG or human IgG. The same isotype control antibody was always included as a negative control, and dead cells were excluded by Annexin V staining (Sigma, USA). Flow cytometry was performed with a 10 color Gallios device (Beckman Coulter, USA), cells were counted using Flow-Count fluorospheres (Beckman Coulter, USA), following the manufacturer’s instructions, and data were analyzed using Flowjo software (Tree Star, Inc, USA).

### Confocal microscopy

FRT tissues were embedded in Tissue-Tek OCT (Sakura, Netherlands). Sections were fixed with acetone, and blocked (50 μg/ml mouse IgG and 10% BSA) and stained with: Ly6g 1A8 (Biolegend), F4/80 BM8 (Biolegend), MHCII 2G9 (BD Biosciences), CD31 2H8 (Invitrogen), α-SMA 1A4 (eBioscience), CD62-E (BD Bioscience), CD62P REA344 (Miltenyi Biotec), ICAM-1 M-19 (Santa Cruz Biotechnology), CD106 429 (Miltenyi Biotec). For protein expression quantification, tissues were imaged using the glycerol ACS APO 20x NA 0.60 objective of a confocal fluorescence microscope (SPE, Leica Microsystems), maintaining the acquisition settings all over the process for each sample and among samples, as previously described (19). Mean fluorescence intensities (MFI) were assessed at multiple regions of interest (ROIs) by field, using the glycerol ACS APO 63x objective and randomly depicted at specific areas of the venular beds in FRT. All quantifications were performed using the FIJI (Fiji Is Just ImageJ) software (NIH).

### Neutrophils depletion

To deplete neutrophils, we administered intravenously 200mg of anti- mouse Ly6G-1A8 (Bio-X-Cell, EE.UU.) in 0,1ml of PBS (17).

### ESR inhibitor and P4 treatment

Female BALB/c were selected in proestrus and injected subcutaneously with 100μl of Faslodex(AstraZeneca) (250mg) to inhibit E2 receptor (ESR) and Prolutex(IBSA) (25mg) as P4 treatment. Then, 12h later, mice were sacrificed and FRT tissues were analyzed by confocal microscopy.

### SELE inhibitor

*In vivo* SELE blocking expression was performed by intravenous injection of 200mg of rat monoclonal anti-mouse SELE antibody (9A9) or control isotype antibody (Bio-X-Cell, USA) in 0.1ml PBS sterile for 12h prior to the experiment (24). Blockade of SELE in endothelial cells was confirmed by confocal microscopy with the anti-mouse CD62-E-REA369 antibody (Miltenyi Biotec).

### Statistical analysis

The test used to determine significance between the treatments in each experiment can be found in the figure legends. GraphPad Prism 5 (GraphPad Software, Inc, USA) was used for statistical analysis. A p value <0.05 was considered statistically significant.

## Results

### Vaginal neutrophil infiltration depends on the ovarian cycle phase in steady-state conditions

**Figure 1:**
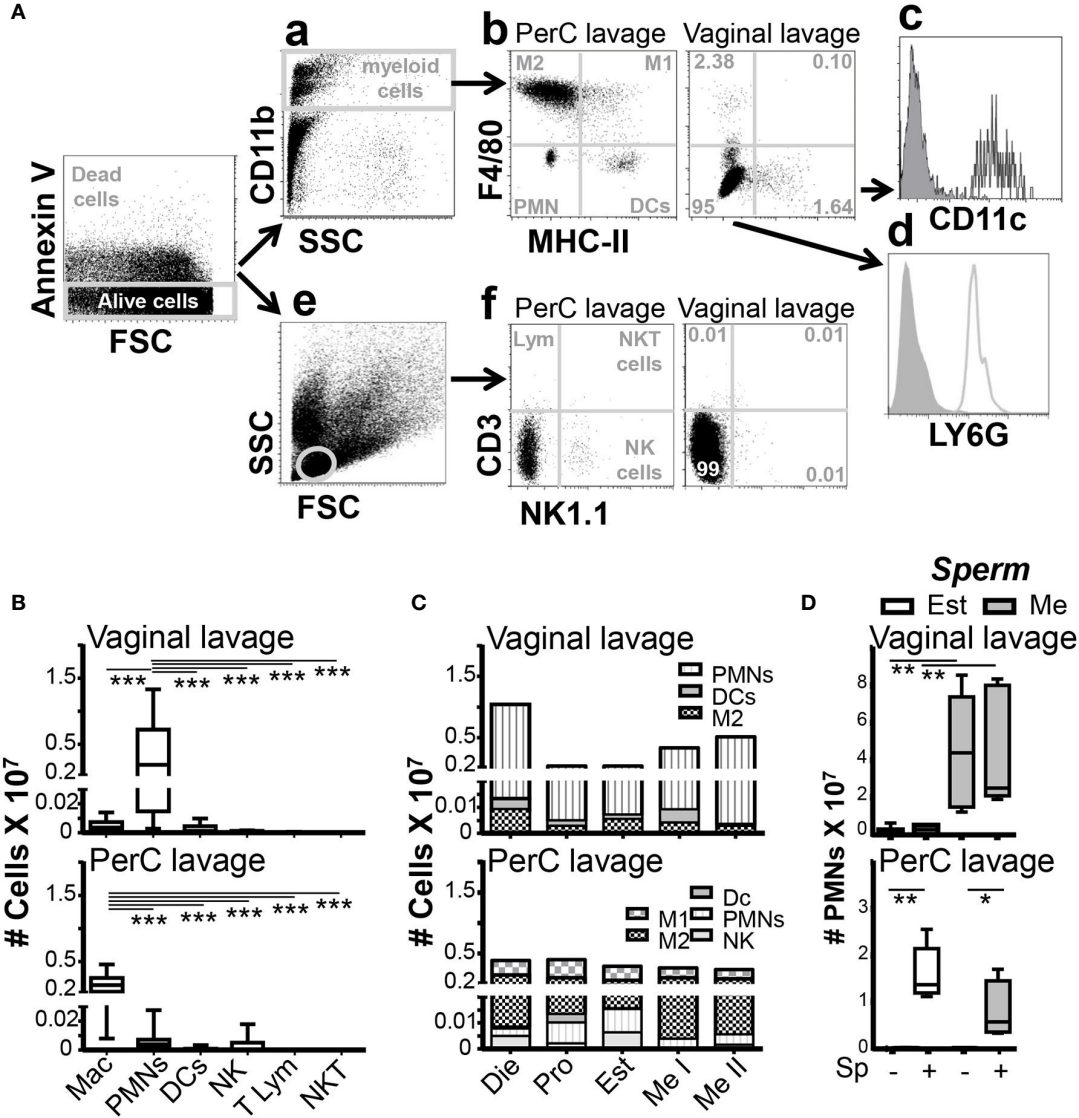
Vaginal lumen immune cells analysis by Flow cytometry. **(A)** Flow cytometry gating strategy. Myeloid analysis: **a**: Dot plot shows a gating region (R1) that identifies myeloid cells based on CD11b positivity and high SSC characteristics. **b**: Plot shows a gate based on f4/80 and MHC-II to sort M1, M2, PMN and DCs. Those populations were further characterized by the markers Ly6G, Ly6C, CD11c, CCR2 (**c** and **d**). Lymphoid analysis: e: Dot plot shows a gating region (R2) that identifies lymphoid cells based on SSC and FSC characteristics. f: Plot shows a gate based on CD3 and NK1.1 to sort lynphocites, NK and NKT cells. **(B)** Identification of distinct immune cells in the vaginal and peritoneal lavage from unstimulated female mice by flow cytometry. Data are expressed as box and whiskers 10-90 percentile (n=8 to 10 mice per group). **(C)** Adult female mice were selected by vaginal smear. Stacked bar chart representation of the immune cells frequency during the ovarian cycle in the peritoneal cavity and the vagina (Data from **Supplemental Figure 1C**). **(D)** Estrus and metestrus adult female mice were selected by vaginal smear and challenged in the vagina or peritoneal cavity with sperm for 2h. Data are expressed as box and whiskers 10-90 percentile (n=8 to 12 mice per group). *p < 0.05, **p < 0.01 and ***p < 0.001. Mann-Whitney test. PerC, peritoneal cavity; Mac, macrophages; M1, inflammatory macrophages; M2, resident macrophages; PMN, neutrophil polymorphonuclears; DC, dendritic cells; NK, natural killers; T Lym, T lymphocytes; NKT, natural killers T cells; Die, Diestrus; Pro, Proestrus; Est, Estrus; Me I, Metestrus I and Me II, Metestrus II.

We studied vaginal lumen immune and peritoneal cells in normal (non-infected) mice by 10 color flow cytometry (**Figure 1A**). We found that neutrophils (CD11B^+^, LY6G^+^, F4/80^-^, CCR2^-^, **Supplementary Figure 1A**; **Supplementary Table 1**) were the predominant immune cells (~95%) in the vaginal lumen, which contrasted with their scarce presence in the bladder and peritoneum (**Figure 1B**; **Supplementary Figure 1B**). Vaginal lumen neutrophils, however, changed in quantity during the menstrual cycle. During proestrus (12 to 20h) light period, neutrophils lower their numbers in the vaginal lumen; however, during estrus (12 to 20h) dark period, neutrophils disappear from the vaginal lumen and slowly start to infiltrate the tissue at the end of that period. Later, during metestrus (12 to 20h) light period again, neutrophils highly infiltrated in the vaginal tissue and lumen (metestrus I ~11-fold, metestrus II ~17-fold). Next, during diestrus stage (2 to 4 days) neutrophil number in the vaginal lumen is very high (~35-fold) (**Figure 1C**; **Supplementary Figure 1C**). Therefore, we used a standard gating strategy (25, 26) that allowed us to determine the vaginal lumen resident immune cells (27). Our results confirmed that, in steady-state conditions, neutrophils are constitutively high recruited to the vaginal lumen, and it was drastically influenced by the ovarian cycle phase (28) and independent of circadian rhythm.

### Vaginal neutrophil influx is independent of insemination

We wondered if sperm challenge could induce neutrophil influx to the vaginal lumen. Notably, we did not detect significant differences between inseminated and non-inseminated mice, number of neutrophils in the vaginal lumen were similarly low during estrus and similarly high during metestrus (**Figure 1D**). To validate the inoculum, we challenged mice with sperm in the peritoneal cavity. The same doses of sperm challenge induced a significant increase in neutrophil influx into the peritoneum in estrus (~200-fold) and metestrus (~80-fold) compared with non-challenged mice (**Figure 1D**). These results revealed that, in contrast to the peritoneum, vaginal lumen neutrophil content was only dependent on the ovarian cycle phase and was not affected by insemination. Our data pointed out that during ovulation, neutrophils disappear from the vagina and sperm do not induce a rapid neutrophil influx.

### Neutrophil influx in the vagina is independent of local microbial aggressions

**Figure 2:**
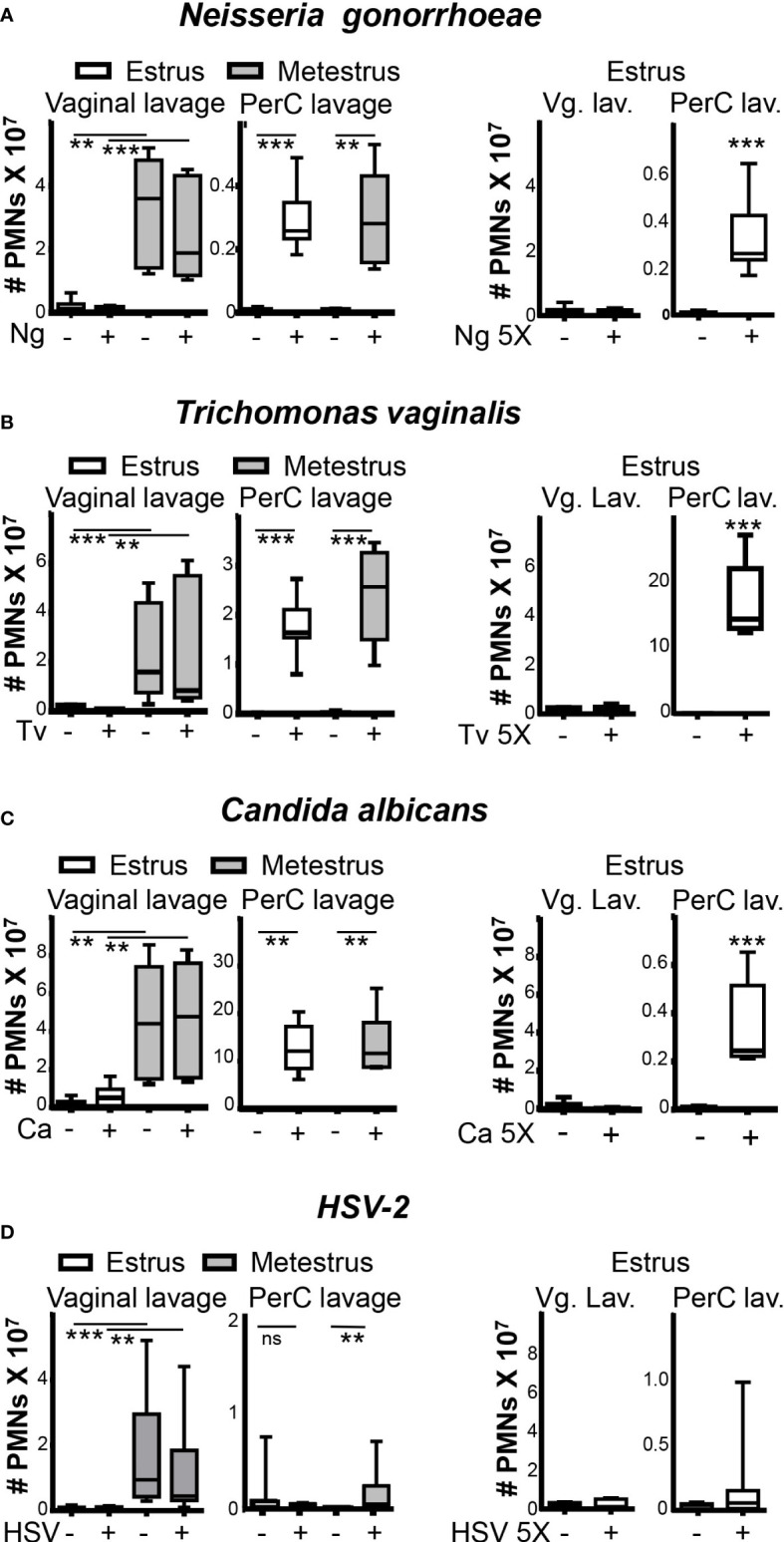
Neutrophils in the vaginal lumen and peritoneal cavity after pathogens challenge. Adult female mice were selected by vaginal smear and challenged in the vagina or peritoneal cavity with **(A)** *Neisseria gonorrhoeae*, **(B)** *Trichomonas vaginalis*, **(C)** *Candida albicans* and **(D)** HSV-2. Number of neutrophils was analyzed by flow cytometry. Data were calculated in at least 3 experiments (n=8 mice per group) and expressed as box and whiskers 10-90 percentile. **p < 0.01 and ***p < 0.001, Mann-Whitney test. PerC, peritoneal cavity; Vg. Lav, Vaginal lavage; Ng, *Neisseria gonorrhoeae*; Tv, *Trichomonas vaginalis* and Ca, *Candida albicans*. ns, non significant.

To test whether pathogens trigger a rapid wave of neutrophil infiltration to the vaginal lumen, we challenged mice with the bacteria *N. gonorrhoeae*, the protozoan *T. vaginalis*, or the fungal pathogen *C. albicans* in the vagina. Two hours later, the number of neutrophils in the vaginal lumen was similarly low during estrus and similarly high during metestrus in the vaginal lumen of challenged and non-challenged mice (**Figures 2A-C**). Of note, the intraperitoneal administration of the same inoculums of *N. gonorrhoeae*, *T. vaginalis*, or *C. albicans* induced significant increases of neutrophils influx in the peritoneum in estrus and metestrus (~40-fold, ~350-fold, and ~200-fold respectively) compared with non-challenged mice. Furthermore, we also failed to detect vaginal neutrophil infiltration during estrus in mice that were vaginally challenged with a five-fold higher concentration of pathogens (**Figures 2A-C**). Our data indicated that the presence of neutrophils in the vagina was only contingent on the ovarian cycle phase and independent of the presence of *N. gonorrhoeae, T. vaginalis*, or *C. albicans*.

We next wondered whether sexually transmitted viruses like HSV-2 induce a differential response. Again, we did not detect a difference in neutrophil numbers in the vaginal lumen between HSV-2 challenged and non-challenged mice (**Figure 2D**), suggesting that pathogens challenge also does not change the ovarian cycle pattern of vaginal leucocyte influx (**Supplementary Figures 2A, B**).

### Estrus prevents neutrophil recruitment into the exocervical tissue

**Figure 3:**
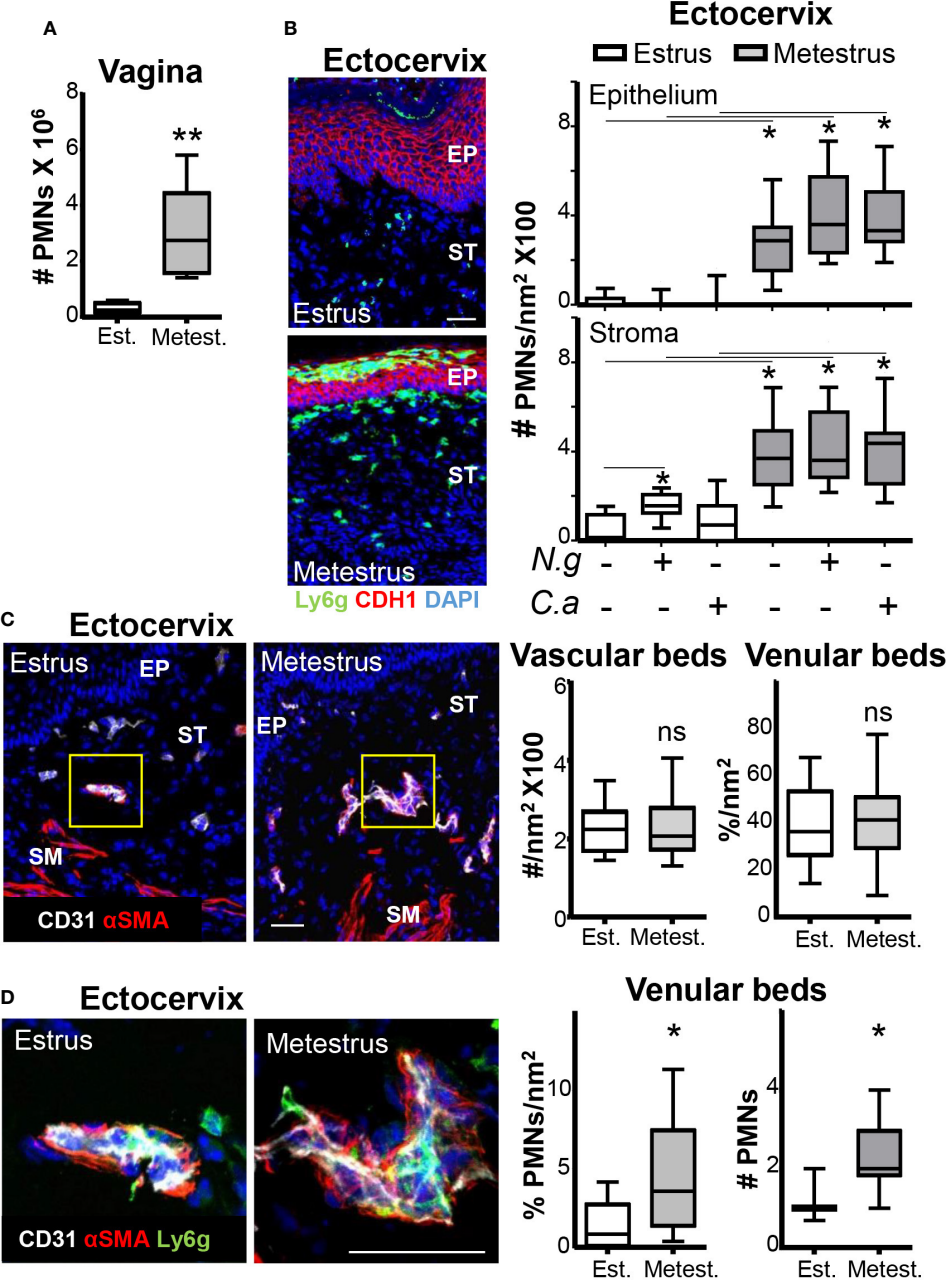
Neutrophils in the ectocervix during the ovarian cycle. Adult female mice were selected by vaginal smear. **(A)** Number of neutrophils in the vaginal tissue by flow cytometry. **(B)** Photomicrograph of the ectocervix of estrus and metestrus mice. Ly6g+ cells (neutrophils) in the cervical epithelium and stroma of estrus and metestrus mice mock or *Neisseria gonorrhoeae* or *Candida albicans* challenged. Data were calculated in at least 3 different sections of each sample. **(C)** Photomicrograph of the vascular and venular beds stained in the ectocervix of estrus and metestrus mice and quantification. **(D)** Quantification of vascular and venular beds neutrophil intraluminal adhesion. Data expressed as box and whiskers 10-90 percentile (n= 5–6 mice per group). *p < 0.05, **p < 0.01 and ns p > 0.05 Mann-Whitney. Scale bar, 50 µm. Est, Estrus; Metest, Metestrus; αSMA, α smooth muscle actin; Ep, epithelium; St, Stroma; Ng, *Neisseria gonorrhoeae* and Ca, *Candida albicans*.

Inhibition of neutrophil influx to the vaginal lumen during estrus could be due to diminished extravasation or impeded transmucosal migration. We analyzed neutrophil numbers in the vaginal tissue in estrus and metestrus mice by flow cytometry and cervix sections to discriminate between these two possibilities (19). We detected low (~10-fold) neutrophil numbers in the vaginal tissues, in the cervical epithelium (~20-fold) and in the stroma (~7-fold) in estrus compared with the respective metestrus samples. Notably, cervical neutrophil numbers in estrus and metestrus were similar in mice infected with *N. gonorrhoeae* or *C. albicans* compared with non-challenged mice (**Figures 3A, B**). These data indicated that the presence of neutrophils in the cervical tissue was also dependent on the ovarian cycle phase and independent of the presence of pathogens. Furthermore, the absence of neutrophil accumulations in the stroma or epithelium during estrus pointed to diminished recruitment from the vascular beds rather than impeded transepithelial migration as the potential operative mechanism. Therefore, we evaluated the number of capillary and venular beds, which were similar in estrus and metestrus cervical tissues (**Figure 3C**). However, the number of attached neutrophils to venular beds in metestrus was higher (~4-fold) than in estrus (**Figure 3D**). These data suggested that estrus diminished the neutrophil recruitment from the vascular beds in the cervix, which was consistent with the lower presence of neutrophils in the vaginal lumen during estrus. Thus, we next focused on the regulation of the neutrophil-endothelial cell (EC) interaction by the menstrual cycle.

### Ovarian cycle regulation of neutrophil adhesion molecule expression on endothelial cells in the vaginal tissue

**Figure 4:**
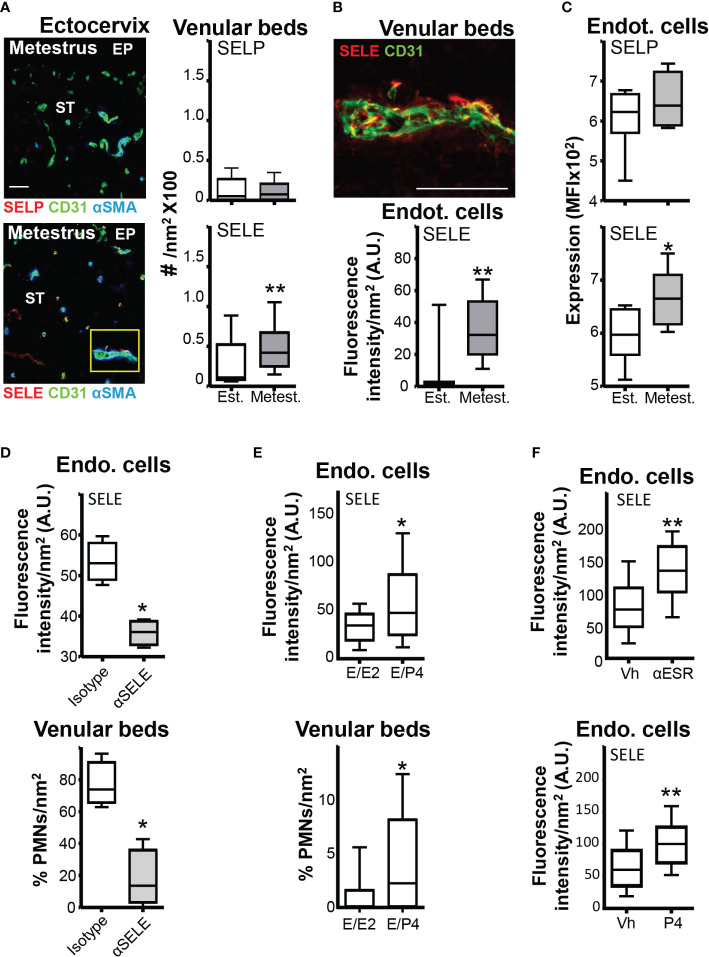
SELE and SELP expression in the ectocervix venular beds. Adult female mice were selected by vaginal smear. **(A)** Photomicrograph of the vascular and venular beds SELP or SELE stained in the ectocervix of estrus and metestrus mice and quantification. **(B)** Photomicrograph of the yellow square in **(A)** Endothelial cells from venular beds SELE stained in the ectocervix of estrus and metestrus mice and quantification of the expression. A and B data were calculated in at least 3 different sections of each sample (n=6 mice per group). **(C)** Flow cytometry average MFI of the SELP or SELE expression in the vaginal venular endothelial cells (n=6 mice per group). **(D)** Anti-SELE treated mice. SELE expression in endothelial cells and quantification of neutrophils intraluminal adhesion in venular beds. **(E)** Hormone-treated ovariectomized mice. SELE expression in endothelial cells and quantification of neutrophils intraluminal adhesion in venular beds. **(F)** Proestrus mice treated with ESR1 inhibitor Faslodex (250 mg) or P4 Prolutex (25 mg). Quantification of SELE expression in endothelial cells in proestrus mice treated with P4 or E2 inhibitor. Data were expressed as box and whiskers 10-90 percentile (n= 5–6 mice per group). *p < 0.05 and **p < 0.01 Mann-Whitney. Est, Estrus; Metest, Metestrus; E2, Estradiol; P4, Progesterona; Vh, Vehicle; Endo. cells, Endothelial cells and A.U., arbitrary units.

The expression of PSGL-1, CD44, CD11a, and CD49d in neutrophils has strong circadian oscillations, resulting in increased efficacy of the leukocyte-endothelium interaction and transmigration process during nighttime (8, 29). We observed that expression of PSGL-1, CD44, CD11a, and CD49d at the same diurnal time (8, 29) in mice was similar in estrus and metestrus (**Supplementary Figure 3A**), suggesting that differential recruitment might be associated with changes in the endothelium. We then evaluated the expression of ICAM1, VCAM1, SELP, and SELE in the EC of the cervical venular beds in estrus and metestrus. As assessed by confocal microscopy and flow cytometry, only SELE showed differential expression in EC of the cervical venular beds (~3-fold higher in metestrus) (**Figures 4A-C**; **Supplementary Figures 3B, C**). Blockade of SELE (24) in metestrus decreased the number of neutrophils in the ectocervical venular beds (**Figure 4D**). Next, we wondered whether SELE differential expression between estrus and metestrus could be due to neutrophil attachment to the venular beds. After depleting neutrophils (11), however, we still detected higher expression of SELE in venular beds in metestrus than in estrus (**Supplementary Figure 3D**). Therefore, we concluded that SELE expression in EC could play a key role in neutrophil extravasation during metestrus, independently of neutrophil attachment.

### Sex hormone regulation of SELE expression on cervical endothelial cells

Our data globally suggested that SELE expression could be a key regulator of cervical neutrophil extravasation during the ovarian cycle. To mimic the ovarian cycle and assess if sex hormones regulated SELE expression in the cervix, we treated ovariectomized mice consecutively with E2 and P4 (17). We detected higher SELE expression (~2-fold) and higher neutrophil numbers in the venular beds of E2/P4-treated mice than in E2/E2-treated ones (**Figure 4E**), suggesting that E2 could down-regulate SELE expression and/or that P4 could promote it. To discriminate between the two possibilities, we treated mice at proestrus with Faslodex (ESR1 inhibitor) or Prolutex (P4) 12 hours before euthanasia. Both treatments increased SELE expression (~2-fold) in the venular ECs during estrus compared with proestrus vehicle-treated mice (**Figure 4F**), suggesting that SELE expression on the venular ECs of the cervix is down-regulated by E2 through the ESR1 during estrus and up-regulated by P4 during metestrus.

## Discussion

In the present study, we show that neutrophil influx to the vagina of mice was exclusively contingent on the ovarian cycle stage, regardless of the presence of infectious agents or sperm. In normal conditions, cervical tissue mediated an exceptionally high and continuous neutrophil infiltrate. During ovulation, however, E2 reduced neutrophil extravasation by down-regulating the expression of SELE on the venular ECs of the cervical tissue, favoring reproduction over the immune defense. After ovulation, P4 up-regulated the expression of SELE and restored immune surveillance. All these ovarian cycle-dependent changes were independent of and not influenced by the presence of various microbial invaders or sperm.

Neutrophils increase in the blood during the day due to a higher release from the bone marrow. At night, circulating neutrophils decrease because they infiltrate tissues (spleen, lung, skin, skeletal muscle, lymph nodes, kidneys, heart, and others) as part of the routine repair, microbiota regulation, or immune surveillance in the absence of inflammatory stimuli (29). Thus, most of the tissues barely express SELP, ICAM-1, or VCAM-1 in the ECs of their venular beds, but their expression peaks in the evening to slightly increase tissue homing of old neutrophils for surveillance, repair, and death (8, 30). Likewise, ECs do not express SELE in normal conditions, but inflammatory signals up-regulate their expression to promote neutrophil extravasation (31–34) and guide neutrophils through ECs (35). Cervical mucosa is a unique site critical for the conciliation of reproductive success and vaginal immunology. Importantly, it must control commensal microbiota and protect against sexually transmitted and opportunistic pathogens that induce infertility (36). In contrast to other tissues, we observed high constitutive expression of SELE, SELP, ICAM1, and VCAM1 in ECs of the cervical venular beds, resulting in exceptionally high and continuous neutrophil infiltration in basal conditions. Therefore, neutrophil infiltration of the vaginal lumen and cervical tissues was independent of circadian rhythms like intestine, liver and white adipose tissue (37). Nevertheless, neutrophils are recruited in large numbers into the vaginal lumen to maintain constant neutrophil immune surveillance against pathogens and to control commensal microbiota balance.

The constitutively high neutrophil content in the vagina could harm sperm quality (11, 12), but the cervical mucosa must allow sperm to swim up during the ovulatory phase of the ovarian cycle. It is well-known that neutrophils do not firmly arrest on ECs that do not express SELE (38). Here, we suggest that, during ovulation, E2 prevents neutrophil entry into the vaginal lumen by down-regulating SELE transcription in venular EC (39) through the E2 receptor (ESR1) because neutrophils do not firmly arrest on EC that do not express SELE (38) to protect sperm from neutrophil attack (11). Then, during the early luteal phase, P4 peaks and up-regulates SELE expression, probably by inhibiting ESR1 effect, and quickly refresh neutrophil influx to restore immunity.

Evaluation of our findings in human tissues is limited due to the lack of an appropriate model to assess infections in a standardized way, as well as to the difficulty of obtaining samples at early stages before the infections are already established. Despite the difference between humans and mice, our results challenge the current universal model of quick and high neutrophil influx to tissues in infections conditions because the neutrophil influx to the vaginal lumen was exclusively dependent on the ovarian cycle phase and high in non-infected mice, except during ovulation. These changes in neutrophil infiltration were not influenced by circadian rhythms or exposure to sperm or infectious agents. During ovulation, neutrophils withdrew from the cervical mucosa to avoid jeopardizing reproductive function. Therefore, basic mucosal protective innate strategies against vaginal STI (40) have evolved to coexist with sperm and to create the estrogen-dependent secretory milieu (41) in the vagina. Clinically, hormonal deregulations may lead to infertility due to the attack of sperm by neutrophils and may compromise vaginal immunity by making it more vulnerable to STDs.

## Data availability statement

The original contributions presented in the study are included in the article/**Supplementary Material**. Further inquiries can be directed to the corresponding authors.

## Ethics statement

The animal study was reviewed and approved by The IiSGM Animal Care and Use Committee and Comunidad de Madrid.

## Author contributions

MR conceived the study. ML, CG-O, IO-V, EB-L, JG, CG-C and PC carried out the experiments. AI-E, MM-F, and PM-R helped with pathogen infection experiments, sample collection key reagents and protocols. ML, FP-M, LS and MR analyzed the data. MR wrote the manuscript and ML, AI-E, MM-F, JV, LS and PM-R revised the manuscript. All the authors approved the submitted version.

## Funding

This work was partially supported by the Ministry of Economy and Competitiveness ISCIII-FIS grants (PI19/00078 and PI19/00132) co-funded by the European Union Funds from the European Commission, “A way of making Europe” and IiSGM intramural grant II-PI-MRC-2017. ML holds IiSGM intramural contract.

## Acknowledgments

Authors thank the units of flow cytometry, confocal microscopy, cell culture, and statistical analysis. We are grateful to J. Villarejo, P. Sanchez-Mateos, F. Asensio and F. Sanchez-Cobos, for expert help and support.

## Conflict of interest

The authors declare that the research was conducted in the absence of any commercial or financial relationships that could be construed as a potential conflict of interest.

## Publisher’s note

All claims expressed in this article are solely those of the authors and do not necessarily represent those of their affiliated organizations, or those of the publisher, the editors and the reviewers. Any product that may be evaluated in this article, or claim that may be made by its manufacturer, is not guaranteed or endorsed by the publisher.

## Supplementary material

Supplementary Figure 1 **(A)** Photomicrograph from light microscopy of the vaginal smear of metestrus mice. Arrow head shows a neutrophil. **(B)** Identification of distinct immune cells in the bladder lavage from unstimulated female mice. **(C)** Adult female mice were selected by vaginal smear. Frequency of the immune cells during the ovarian cycle in the peritoneal cavity and the vagina lavage. Data are expressed as box and whiskers 10-90 percentile (n=8 to 10 mice per group). *p<0.05. Mann-Whitney. PerC, peritoneal cavity; M1, inflammatory macrophages; M2, resident macrophages; PMNs, polymorphonuclears; DC, dendritic cells; NK, natural killers; T Lym, T lymphocytes; Die, Diestrus; Pro, Proestrus; Est, Estrus; Me I, Metestrus I and Me II, Metestrus II

Supplementary Figure 2 Adult female mice were selected by vaginal smear and challenged in the vagina or peritoneal cavity with **(A)** *Neisseria gonorrhoeae*, and **(B)** HSV-2. Number of cells was analyzed by flow cytometry. Data were calculated in at least 3 experiments (n=8 mice per group) and expressed as box and whiskers 10-90 percentile. *p>0.05, **p<0.01 and ***p<0.001, Mann-Whitney. PerC, peritoneal cavity; Ng, *Neisseria gonorrhoeae* and Sp, sperm.

Supplementary Figure 3 Adult female mice were selected by vaginal smear. **(A)** Flow cytometry analysis of PSGL-1, CD11A, CD44 and CD49d expression in blood neutrophils in estrus and metestrus mice. **(B)** Confocal microscopy photomicrograph of the venular beds and flow cytometry analysis of estrus and metestrus mice stained with VCAM and **(C)** ICAM-1. **(D)** SELE expression in neutrophil depleted mice (anti-Ly6g IV delivery). Confocal microscopy analysis of SELE expression in the venular beds of estrus and metestrus mice. Data were expressed as box and whiskers 10-90 percentile (n= 5–6 mice per group). **p<0.01 Mann-Whitney. Scale bar, 50 µm. Ep, epithelium; St, Stroma; Est, Estrus; Metest, Metestrus; Endo. cells, Endothelial cells and A.U., arbitrary units.

Supplementary Table 1 Summary of the markers used to define the cells population studied.
