# Supplementary figures and images for: Vaginal neutrophil infiltration is contingent on ovarian cycle phase and independent of pathogen infection
Source: Front Immunol. 2022 Dec 8;13:1031941. doi: 10.3389/fimmu.2022.1031941 (PMC9771706; doi:10.3389/fimmu.2022.1031941)

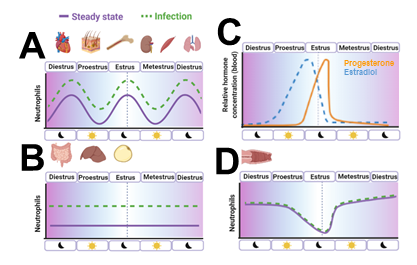

Supplement: Supplementary Figure 1 — (A) Photomicrograph from light microscopy of the vaginal smear of metestrus mice. Arrow head shows a neutrophil. (B) Identification of distinct immune cells in the bladder lavage from unstimulated female mice. (C) Adult female mice were selected by vaginal smear. Frequency of the immune cells during the ovarian cycle in the peritoneal cavity and the vagina lavage. Data are expressed as box and whiskers 10-90 percentile (n=8 to 10 mice per group). *p<0.05. Mann-Whitney. PerC, peritoneal cavity; M1, inflammatory macrophages; M2, resident macrophages; PMNs, polymorphonuclears; DC, dendritic cells; NK, natural killers; T Lym, T lymphocytes; Die, Diestrus; Pro, Proestrus; Est, Estrus; Me I, Metestrus I and Me II, Metestrus II [file Image_1.tif]

Supp.table1

|       | Mac-M1 | Mac-M2 | PMNs | CDs | NK | T Lyn | NKT |
|-------|--------|--------|------|-----|----|-------|-----|
| Cd11b | +      | +      | +    | +   |    |       |     |
| Ly6G  | -      | -      | +    | +   |    |       |     |
| Ly6C  | +      | -      | +    | +   |    |       |     |
| F4/80 | +      | +      | -    | -   |    |       |     |
| CCR2  | +      | -      | -    | +   |    |       |     |
| CCR3  | -      | -      | -    | +   |    |       |     |
| CD244 | +      | +      | -    | +   |    |       |     |
| MHCII | +      | -      | -    | -   |    |       |     |
| CD3   |        |        |      |     | +  | -     | +   |
| NK1.1 |        |        |      |     | -  | +     | +   |

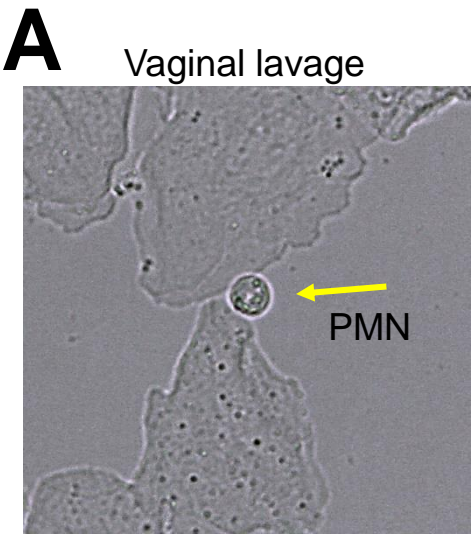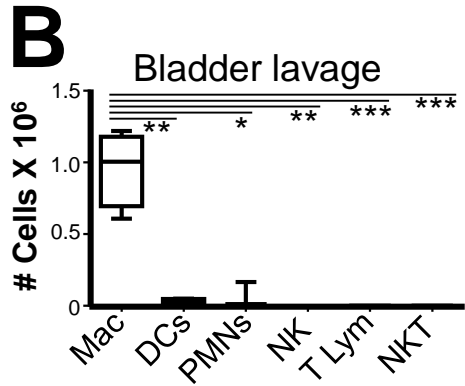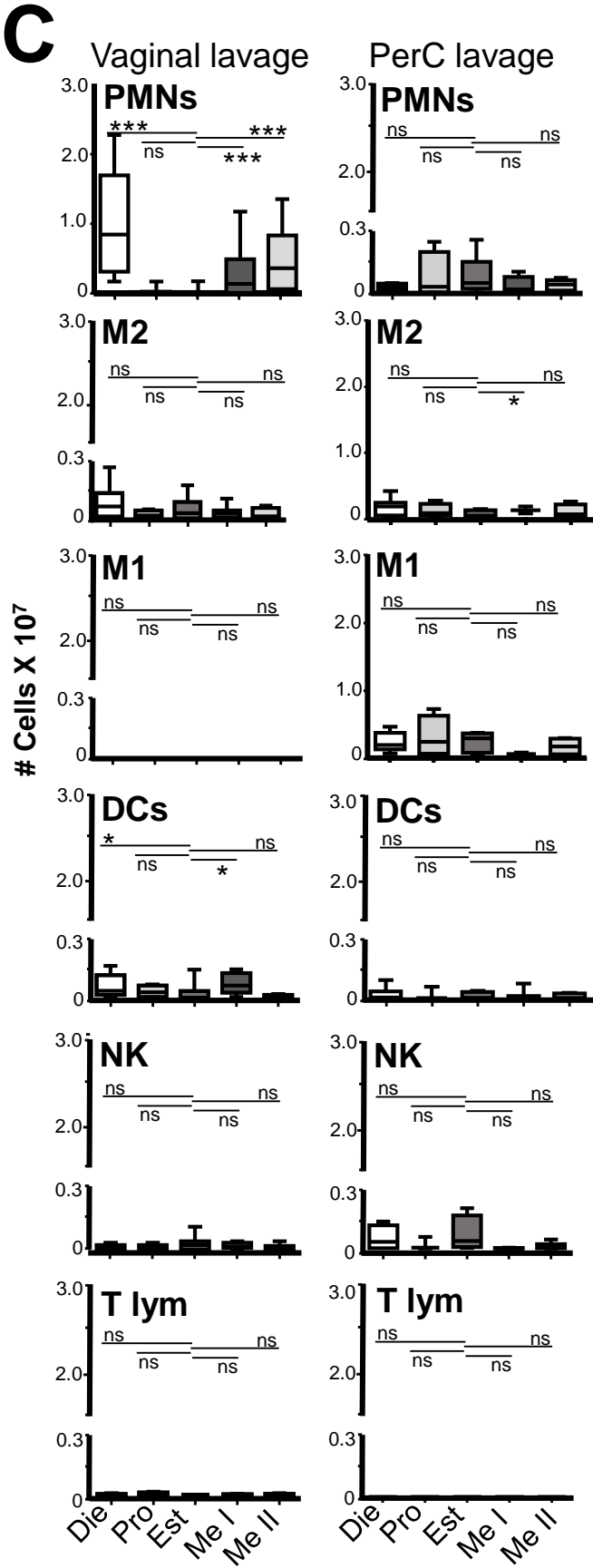

Supp.Fig. 2

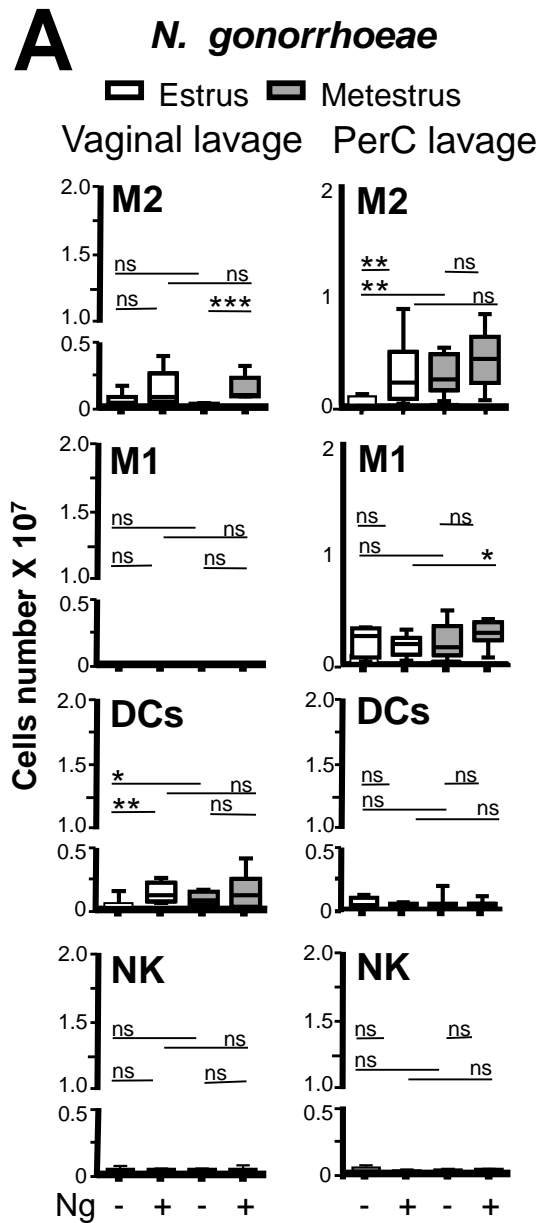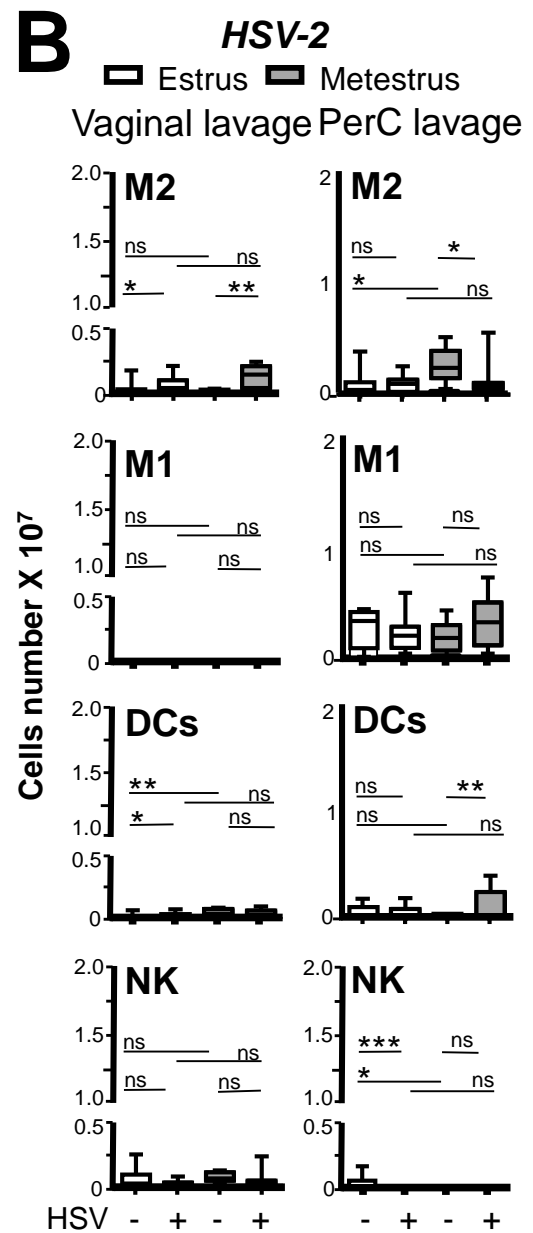

Supp.Fig. 3

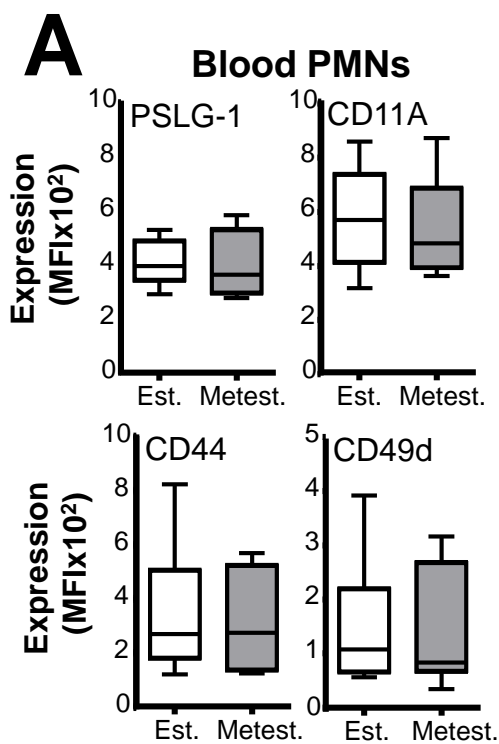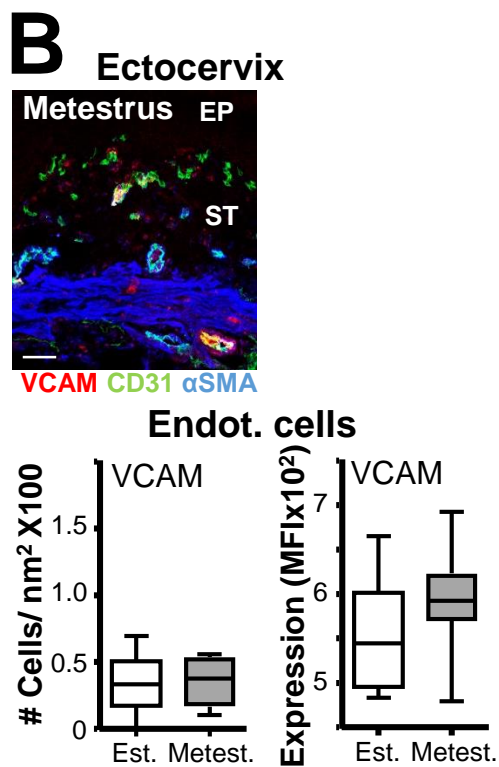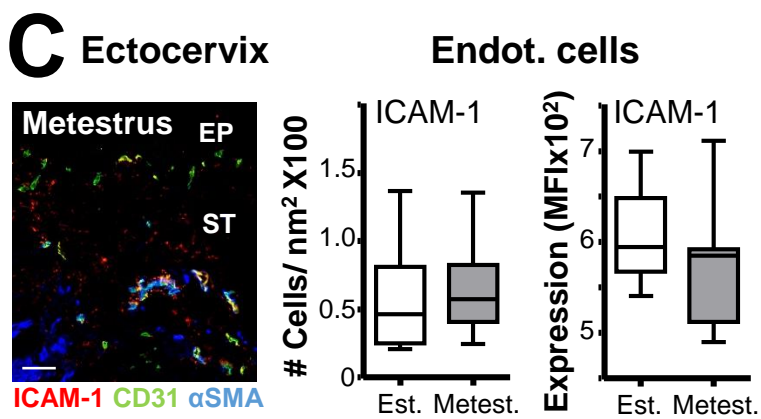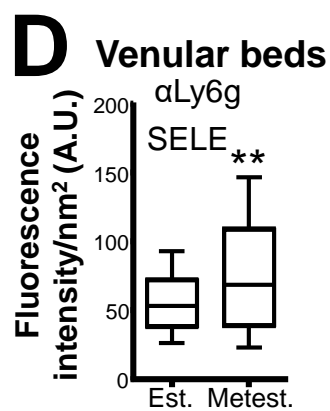

Supplement: Supplementary Figure 2 — Adult female mice were selected by vaginal smear and challenged in the vagina or peritoneal cavity with (A) Neisseria gonorrhoeae, and (B) HSV-2. Number of cells was analyzed by flow cytometry. Data were calculated in at least 3 experiments (n=8 mice per group) and expressed as box and whiskers 10-90 percentile. *p>0.05, **p<0.01 and ***p<0.001, Mann-Whitney. PerC, peritoneal cavity; Ng, Neisseria gonorrhoeae and Sp, sperm. [file DataSheet_1.pdf]
